# Persistence to Multiple Myeloma Treatments Given at First Relapse—Results From the Australian Real‐World OPTIMISE Study

**DOI:** 10.1002/jha2.70377

**Published:** 2026-08-21

**Authors:** Felix Buescher, Steve Su, Ben Waterhouse, Cletus Pinto

**Affiliations:** ^1^ Janssen‐Cilag Pty Ltd. Sydney Australia; ^2^ Model Solutions Pty Ltd. Sydney Australia

**Keywords:** daratumumab, real‐world evidence, relapsed refractory multiple myeloma, second‐line treatment, treatment persistence

## Abstract

**Background:**

Daratumumab became publicly available in Australia in 2021 in combination with bortezomib and dexamethasone (DVd), as a second‐line (2L) therapy for multiple myeloma (MM). We evaluated real‐world persistence to any MM treatment after first relapse before and after DVd availability using the Pharmaceutical Benefits Scheme 10% sample dataset.

**Methods:**

Dispensing records for adults receiving 2L MM treatment from January 2018 to December 2020 (Period 1) and January 2021 to April 2025 (Period 2) were analyzed using Kaplan–Meier to determine treatment persistence.

**Results:**

Eighty patients were included in Period 1 and 290 in Period 2. Median treatment persistence was longer in Period 2 (15 months) than Period 1 (9.8 months) (hazard ratios [HRs]: 0.46; 99.9% CI: 0.23–0.90; *p* < 0.001). Treatment persistence in patients who progressed within 18 months on first‐line therapy was longer in Period 2 (13 months) than Period 1 (9.2 months) (HR: 0.53; 95% CI: 0.34–0.81; *p* < 0.01). The Period 2 lenalidomide‐refractory cohort (*n* = 78) demonstrated a median persistence of 11 months (95% CI: 8.3–23 months). The number of lenalidomide‐refractory patients in Period 1 (*n* = 11) was insufficient for comparative analysis.

**Conclusions:**

The observed improvements in treatment persistence among patients with MM including those refractory to lenalidomide, following reimbursement‐driven availability of DVd, indicate meaningful clinical benefit in routine practice after the introduction of novel therapies, and underscores the need for sustained access to diverse and effective treatment options for relapsed MM.

**Trial Registration**: The authors have confirmed clinical trial registration is not needed for this submission.

## 1 Background

Multiple myeloma (MM) is the second most common hematological malignancy, and its incidence and prevalence are expected to continue rising [1]. Australia has among the highest global MM incidence and mortality rates [2], with an incidence of 8.7 per 100,000 population, which is projected to increase to 10.0 per 100,000 population by 2043 [2]. In contrast, the age standardized MM mortality rate in Australia is projected to decline by 27.5% between 2018 and 2043, from 4.0 to 2.9 cases per 100,000 population [2]. This is largely attributed to earlier diagnosis, clinical management, and advances in MM treatment, which include immunotherapies such as bispecific antibodies and cellular therapies [3].

Despite this progress, an estimated 16% of newly diagnosed MM patients have primary refractory disease and almost all responding patients eventually relapse [4, 5, 6]. In Australia, the main second‐line (2L) treatment options are combination therapies including immunomodulatory drugs (thalidomide, lenalidomide, and pomalidomide), proteasome inhibitors (bortezomib and carfilzomib), an anti‐CD38 monoclonal antibody (daratumumab), alkylating agents, anthracyclines, and corticosteroids.

In Australia, daratumumab was listed in the Pharmaceutical Benefits Scheme (PBS) in January 2021 in combination with bortezomib and dexamethasone (DVd), for patients with relapsed or refractory MM (RRMM) who have received one prior line of therapy. Patients with RRMM who had one prior line of therapy benefited the most from DVd therapy in the Phase 3 CASTOR trial. In the CASTOR trial, adding daratumumab to bortezomib and dexamethasone (Vd) significantly prolonged progression‐free survival (PFS) and overall survival (OS) in RRMM compared to Vd alone (patients with one prior line of therapy PFS: median 27.0 vs. 7.9 months, hazard ratio [HR], 0.22, 95% CI, 0.15–0.32, *p* < 0.001; OS after median 72.6 months [range: 0.0–79.8 months] follow up: not estimable median range versus 47.0 months, HR: 0.56, 95% CI: 0.39–0.80) [7, 8, 9].

The treatment effects observed in clinical trials have been evaluated in routine clinical practice, using treatment persistence (time to treatment discontinuation) and time to next treatment (TTNT), as proxy measures of PFS [10, 11]. Clinical trials assess therapeutic efficacy under ideal conditions with strict patient selection, and study designs often unfeasible in real‐world settings, while real‐world evidence (RWE) demonstrate the benefits of the treatment as used in routine practice [12]. Discrepancies often exist between the effectiveness of an intervention reported in clinical trials and observed in real‐world studies [12].

A real‐world multicenter study in the United Kingdom examined the overall response rate (ORR) and PFS of DVd treatment in 296 patients with MM who had previously received one line (1L) of treatment [13]. After a 21 month median follow up, ORR was 82%, median PFS was 16 months (95% CI: 12–20 months) and the median OS was not reached [13]. This study showed that patients with MM benefited from DVd, but the PFS was shorter than the PFS from the CASTOR trial, likely due to factors such as differences in patient demographics [13]. Other real‐world studies evaluating DVd treatment in RRMM also reported different outcomes compared to those observed in the CASTOR trial [14, 15]. These studies highlight the value of RWE as it reflects the effectiveness of a therapy observed in routine clinical settings beyond the limited environment of clinical trials [13].

Based on previous RWE studies, patient demographics and clinical context can significantly impact treatment effectiveness. Therefore, using RWE is important to better understand what is happening in clinical practice. There are currently no published RWE studies from Australia that examine MM treatment persistence. The current OPTIMISE study addresses this gap by evaluating real‐world to 2L therapy persistence in Australian patients with MM before and after the introduction of DVd. This retrospective observational study used the Australian Commonwealth Department of Human Services PBS 10% sample dataset [16] (a longitudinal, de‐identified prescription claims database), to analyze treatment patterns, outcomes, and the impact of reimbursement‐driven access to novel therapies [17]. The primary objective was to compare persistence to 2L therapies in two cohorts: pre‐DVd (January 2018–December 2020) and post‐DVd availability (January 2021–April 2025). Secondary objectives included identifying factors influencing persistence, such as early progression, lenalidomide refractoriness, and demographics. This study is the first to evaluate the population‐level impact of reimbursement‐driven DVd access on treatment persistence in a nationally representative Australian cohort.

## 2 Methods

### 2.1 Data Collection

This was a retrospective observational study of RWE from the Australian PBS 10% sample, which was considered representative of the Australian population, and included dispensing data from prescription medicines in Australia. It did not capture dispensing of private prescriptions or medicines supplied to patients in public hospitals [16]. The PBS 10% dataset used in this study was analyzed by Model Solutions Pty Ltd. (the data custodian) and included patients prescribed MM treatments in Australia from January 2018 to April 2025. The study was approved by the Australian Government Department of Human Services External Request Evaluation Committee (RMS4613).

Extracted data were from patients aged 18 years or older at the time of first prescription of any treatments of interest. Included patients had been dispensed as a 1L and a 2L MM therapy (Table 1). To remove non‐responders and patients who did not tolerate treatment, patients were required to have persisted on the prescribed treatment drug of interest for a minimum of 3 months. All patients who had dispensing records for daratumumab were included in the study as, at the time of extraction, it was only available for 2L treatment of MM in Australia. It was assumed that patients with no 1L dispensing record had received their 1L treatment in a clinical trial or other non‐PBS source.

**TABLE 1:** Therapies available in Australia for treatment of first‐ and second‐line multiple myeloma.

1L treatments	2L treatments	
	January 2018–December 2020 (Period 1)	January 2021–April 2025 (Period 2)
Bortezomib, lenalidomide, dexamethasone (VRd)	Carfilzomib, dexamethasone (Kd)	Carfilzomib, dexamethasone (Kd)
Bortezomib, cyclophosphamide, dexamethasone (VCd)	Pomalidomide, dexamethasone (Pd)	Pomalidomide, dexamethasone (Pd)
Lenalidomide, dexamethasone (Rd)	Bortezomib, dexamethasone (Vd)	Bortezomib, dexamethasone (Vd)
Bortezomib, dexamethasone (Vd)		Daratumumab, bortezomib, dexamethasone (DVd)

Records were obtained from January 2018 to December 2020 (Period 1), prior to the listing of DVd, and contained information from patients taking carfilzomib (Kyprolis)‐dexamethasone (Kd), pomalidomide (Pomalyst)‐dexamethasone (Pd), and Vd (Velcade) (Table 1). Following the PBS listing of DVd, dispensing records were obtained from January 2021 to April 2025 (Period 2) containing information from patients taking Kd, Pd, Vd, or DVd (Table 1). The unequal observation periods reflect the timing of DVd reimbursement and availability of data at extraction; sensitivity analyses were not expected to be materially affected by period length. To ensure similarity between the two groups, other regimens available in Australia that had low to no patients at 2L, such as the combinations pomalidomide, bortezomib, and dexamethasone (PVd), and elotuzumab, lenalidomide, and dexamethasone (Eld) were excluded (Table 2). Due to difficulties in interpreting the PBS item codes, patients who received lenalidomide for 2L treatment were excluded from this study.

**TABLE 2:** Patient demographics and treatment characteristics.

	January 2018–December 2020 (Period 1)	January 2021–April 2025 (Period 2)
Number of patients (*n*)	80	290
Gender (*n*, % male)	52 (65%)	164 (57%)
Age at initiation, years, *n* (%)		
< 60	16 (20%)	42 (14%)
60–69	13 (16%)	59 (20%)
70–79	35 (44%)	118 (41%)
80–89	16 (20%)	66 (23%)
≥ 90	0 (0%)	5 (2%)
Early progressors (*n*, %)	28 (35%)	120 (41%)
Lenalidomide refractory (*n*, %)	11 (14%)	78 (27%)
First‐line therapy		
V‐based	20 (25%)	75 (26%)
VR‐based	2 (3%)	110 (38%)
R‐based	13 (16%)	49 (17%)
Other	45 (56%)	56 (19%)
Second‐line therapy		
Kd	40 (50%)	17 (6%)
Pd	2 (3%)	4 (1%)
Vd	38 (48%)	14 (6%)
DVd	0 (0%)	255 (89%)

*Note*: R = lenalidomide; V = bortezomib.

The data were extracted on June 18, 2025, and included gender, year of birth, date of claim, year of death, and PBS item code. The variables of interest were time since first MM treatment (defined in years since the first prescription of a treatment prescribed for MM), time of subsequent MM treatment, type of 2L therapy received, early progressor status, lenalidomide‐refractory status, age group (< 60, 60–69, 70–79, 80–89, ≥ 90), and gender.

Early progressors were defined as patients who were no longer dispensed 1L therapy (bortezomib‐based, lenalidomide‐based, bortezomib‐lenalidomide combination‐based) within 18 months of the initial dispensing record for the therapy. Lenalidomide‐refractory patients were defined as those with a dispensing record for a subsequent MM treatment within 60 days of the date of the last dispensing record for lenalidomide (either lenalidomide‐based [R‐based] or bortezomib‐lenalidomide combination‐based [VR‐based]). Type of 1L therapy received was categorized as bortezomib‐based (V‐based), R‐based, VR‐based, and other.

Treatment duration was defined as the time in consecutive days between the first dispensing record for one of the treatments of interest to the discontinuation date, death or the last date of data extraction, depending on which occurred first. Patients were censored at the last data extraction or death. Treatment discontinuation was defined as a ≥ 6‐month gap without dispensing.

### 2.2 Data Analysis

Baseline demographic data were summarized descriptively. Kaplan–Meier (KM) survival curves were generated to visualize treatment persistence, and the log‐rank test was used to compare KM curves between treatment groups. Cox proportional hazard models were employed to estimate HRs and 95% confidence intervals (CIs), adjusting for covariates such as age, gender, 1L therapy, early progression, and lenalidomide‐refractory status. Restricted mean survival time (RMST) analyses were conducted to account for non‐proportional hazards where applicable. Bonferroni adjustments were applied to control the family‐wise error rate at 0.05 across 50 hypothesis tests (RMST and HR *p*‐values combined). Accordingly, a result is considered significant if *p* < 0.001 (i.e., 0.05/50). As a result, all inferential analyses used two‐sided tests with a 0.1% significance level, corresponding to 99.9% CIs unless otherwise stated. Statistical analyses were conducted using the statistical software R.

## 3 Results

### 3.1 Patient Demographics

In the PBS 10% dataset, there were 80 patients over 18 years with MM who were prescribed Kd, Pd, or Vd as their 2L therapy in Period 1 (Table 2). A total of 290 patients with MM were prescribed Kd, Pd, Vd, or DVd as their 2L therapy Period 2. Most patients in Period 2 received DVd as their 2L therapy (89%).

Patient demographics for 2L therapy were similar across the two time periods; however, patients were twice as likely to be lenalidomide‐refractory in Period 2 compared to Period 1 (27% vs. 14%). This was likely driven by timing of reimbursed access to 1L lenalidomide in Australia, as both 1L lenalidomide maintenance and bortezomib, lenalidomide, dexamethasone (VRd) were listed on the PBS in 2020. The 1L therapy was more often VR‐based in Period 2 compared to Period 1 (38% vs. 3%). Despite a higher‐risk population in Period 2, characterized by a greater proportion of lenalidomide‐refractory patients, persistence outcomes were improved, consistent with an effect associated with treatment access rather than by population shift alone.

### 3.2 Duration of Treatment

**FIGURE 1:**
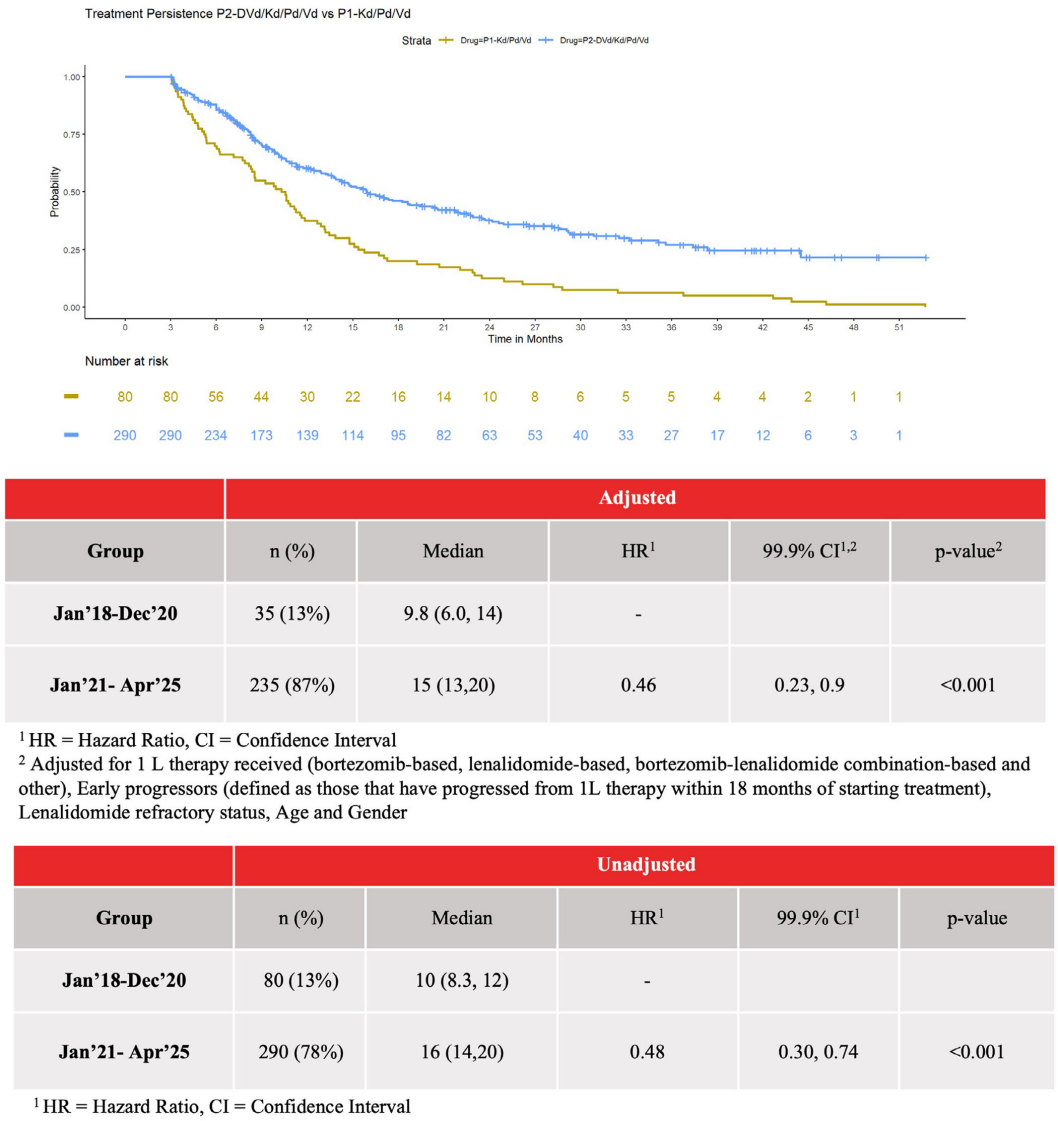
Kaplan–Meier estimate of persistence to treatment in Period 2 compared to Period 1.

**FIGURE 2:**
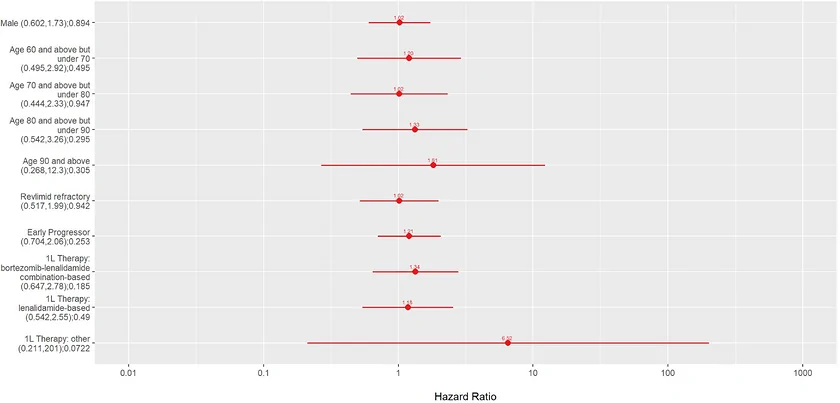
Treatment persistence in Period 2 compared to Period 1 by subgroups.

Treatment duration with 2L MM therapies was significantly longer in Period 2 following the DVd availability than in Period 1 (Figure 1). The median persistence in Period 2 was 15 months (95% CI: 13–20 months) versus 9.8 months (95% CI: 6.0–14 months) in Period 1 (HR: 0.46, 99.9% CI: 0.23–0.90; *p* < 0.001). KM survival curves demonstrated a clear separation between the two cohorts, favoring Period 2. This was seen in both unadjusted (HR: 0.48 [99.9% CI: 0.3–0.74], *p* < 0.001) and adjusted analyses (HR: 0.46 [99.9% CI: 0.23–0.9], *p* < 0.001) (Figure 1B,C). The adjusted Cox analysis of treatment duration found that none of the assessed covariates affected the duration of treatment (Figure 2). The PBS recommends a fixed duration (11 cycles) of Vd, which may have affected treatment persistence in Period 1 and to a lesser degree Period 2 (see Section 4.1).

### 3.3 Persistence in Early Progressors and Lenalidomide‐Refractory Patients

**FIGURE 3:**
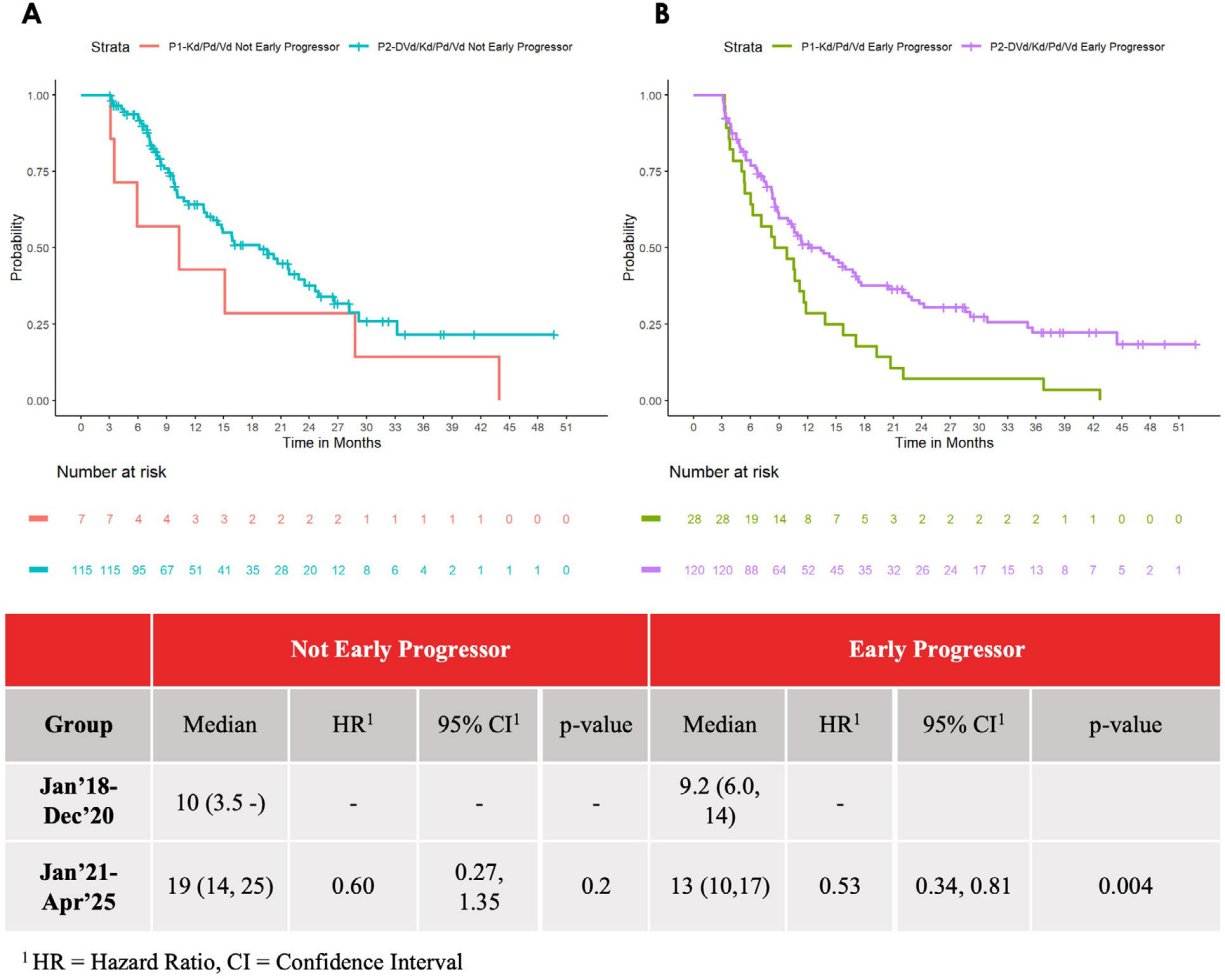
Kaplan–Meier estimate of persistence to treatment in Period 2 compared to Period 1 for patients by early progressor status (A) not early progressor and (B) early progressor.

**FIGURE 4:**
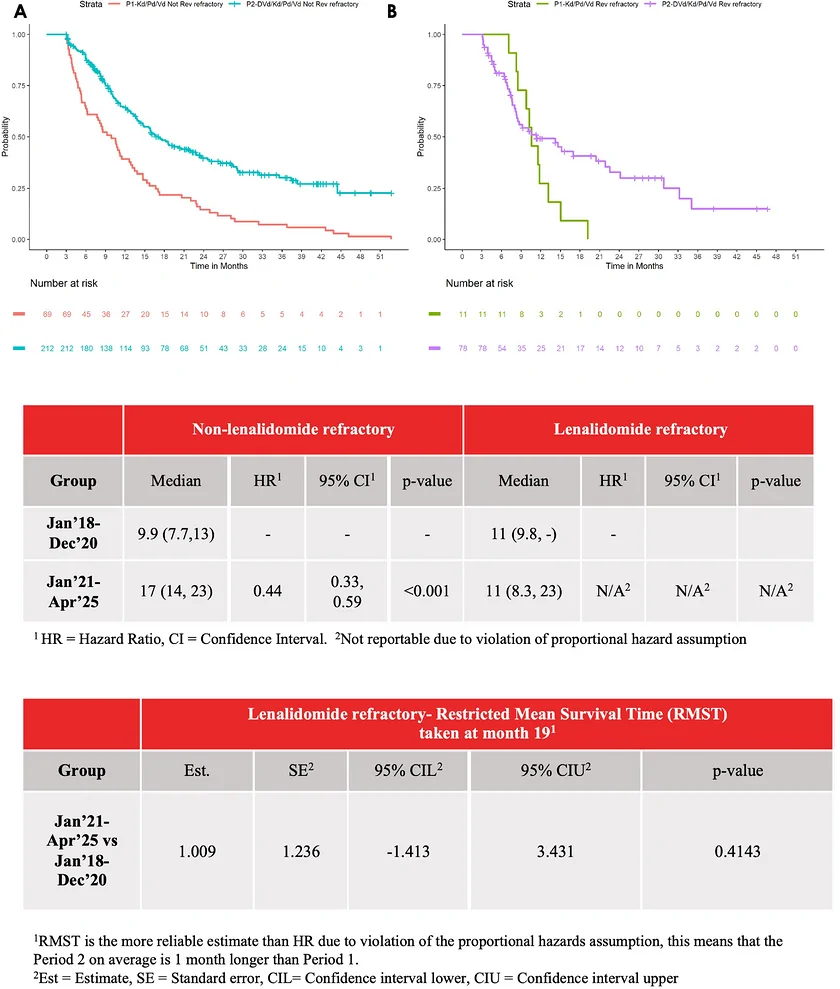
Kaplan–Meier estimate of persistence to treatment in Period 2 compared to Period 1 by lenalidomide‐refractory status (A) non‐lenalidomide refractory and (B) lenalidomide refractory.

Among early progressors, median persistence was longer in Period 2 (13 months; 95% CI: 10–17 months) than Period 1 (9.2 months; 95% CI: 6.0–14 months), (HR: 0.53, 95% CI: 0.34–0.81, *p* = 0.004) (Figure 3). Although based on a small sample size, lenalidomide‐refractory patients in Period 2 had a median persistence of 11 months (95% CI: 8.3–23 months). The small number of lenalidomide‐refractory patients in Period 1 made comparison with Period 2 difficult (Figure 4). As proportional hazards assumptions were not met for this subgroup, RMST was used as the primary comparative measure instead of HR (Figure 4B).

## 4 Discussion

The MM treatment landscape is constantly evolving; however, understanding the impact of reimbursement‐driven access to active regimens remains both clinically and policy relevant. Treatment persistence (or duration of therapy), is a useful proxy measure for PFS, providing a pragmatic measure of treatment use in real‐world datasets where direct clinical endpoints such as PFS are unavailable [10, 11, 18, 19].

Evaluating Australian PBS dispensing data is a common approach used in many RWE studies [17, 20]. Our study showed a large uptake in the use of DVd once it had become available on the PBS. This demonstrates the need for investment in novel MM therapies with new mechanisms of action. Our study also showed 2L MM therapy persistence increased after DVd became available in Australia. Results remained consistent across adjusted analyses including gender, age, early progressors, or prior 1L treatment. Despite the small cohort, the public availability of DVd showed a meaningful benefit among lenalidomide‐refractory patients, a growing yet difficult to treat patient cohort [21]. This finding is particularly relevant in the contemporary treatment landscape, where lenalidomide‐refractory disease is increasingly common. Our study aligns with previous RWE showing high PFS rates in patients with MM prescribed daratumumab as a 2L monotherapy or in combination [13, 14, 15]. Although this study did not specifically assess it, the increased treatment persistence observed following the PBS listing of DVd may contribute to better outcomes and potentially longer survival for patients with MM in Australia.

The persistence observed following DVd availability in this Australian cohort is aligns with UK and European RWE. The median persistence in this study was 15 months (95% CI: 13–20 months), aligning with UK and Italian RWE studies evaluating DVd treatment in lenalidomide exposed RRMM patients, where median PFS was 16 and 17 months, respectively [13, 14]. A Slovakian retrospective multicenter study evaluated DVd treatment in RRMM patients refractory to at least one line of treatment reported a slightly lower median PFS of 10 months compared our study [15]. Another UK RWE study examined DVd as 2L in a similar patient population to our study (RRMM patients exposed to lenalidomide, refractory to lenalidomide, or neither), reported a median time to treatment discontinuation or death of 11.9 (11.0–12.7) months and median TTNT or death of 17.4 (16.3–18.7) months [22]. Our study expands existing RWE by evaluating impact of DVd access in an Australian population. As RWE includes patients with broad clinical characteristics, ethnicities, and comorbidities, so the treatment persistence differences observed in Australia, the United Kingdom, Italy, and Slovakia may reflect this diversity. Notably, early progressors in the Period 2 showed longer median persistence than Period 1, highlighting the need for continued access to novel treatments for patients with aggressive disease progression.

There is a clinical need for effective 2L treatments for lenalidomide‐refractory patients, who have poor prognosis and represent a growing proportion of the relapsed population due to the widespread use of 1L lenalidomide, including in the post‐stem cell transplant maintenance setting [21]. The CASTOR trial enrolled a small population of lenalidomide‐refractory patients who received DVd, reporting a median PFS of 7.8 months in the intention‐to‐treat population [7]. Our analysis, although also based on a small population showed that lenalidomide‐refractory patients had a median persistence of 11 months after the availability of DVd. There was a higher proportion of lenalidomide‐refractory patients in Period 2 (55%) compared to Period 1 (19%). Remarkably, despite a higher proportion of lenalidomide‐refractory patients in Period 2, overall persistence remained superior to Period 1.

Our findings align with a previous real‐world dataset where DVd treatment in lenalidomide‐refractory patients had a median PFS of 12.9 months in Canada [23]. Similarly, an Italian retrospective study reported a median time to disease progression of 10.8 months, with 1‐ and 2‐year TTDP rates of 44.7% and 25.3%, respectively [24]. Another Italian retrospective study reported a median PFS of 14 months and median TTNT of 20 month in lenalidomide‐refractory patients treated with DVd at first relapse [25]. These RWE studies demonstrate that DVd may be an effective treatment option for this hard‐to‐treat heterogeneous patient population.

### 4.1 Strength and Limitations

The PBS 10% dataset allowed us to evaluate the persistence to therapy in a nationally representative, real‐world population, and avoiding selection bias that may occur in smaller datasets.

The PBS 10% dataset does not provide clinical details on disease severity, polypharmacy, or cytogenetic risks; thus, surrogates are determined through analyzing the data, such as line of therapy and time since first MM agent dispensing. Due to the absence of a date of death, the analysis uses the earliest possible date of death. This means that medication use could be underestimated among those who died. The PBS 10% data also does not identify patients who were not treated, such as those who filled their prescription but did not take the medicine. The PBS used multiple item codes for lenalidomide and its combinations, so it was not possible to distinguish whether lenalidomide was dispensed as 2L treatment or posttransplant maintenance. To account for uncertainty, patients receiving Rd for reasons other than 1L treatment, were excluded from the study. In addition, the PBS dataset is claims‐based and does not differentiate between reasons for treatment discontinuation, such as toxicity, disease progression, death, or patient preference. As such, persistence should therefore be interpreted as a pragmatic utilization endpoint.

A significant proportion of 2L therapy in Period 1 consists of Vd. The PBS recommended maximum duration of Vd treatment is 11 cycles (approximately 7.7 months). Although Vd has a fixed duration, our data show that nearly half the patients were still able to continue Vd therapy beyond 11 cycles (Figure S1). This suggests that the decision to discontinue Vd treatment was likely influenced by clinical judgment such as treatment efficacy and tolerability rather than by its fixed duration. However, it is possible that this fixed duration may have impacted treatment duration in both periods.

Despite these limitations, this dataset remains an important source of information representative of the use of medications across the whole Australian population and is widely referenced in many RWE studies [16, 17, 20].

In addition, the patient for Period 1 not early progressors and lenalidomide‐refractory was very low. This hindered analysis between Periods 1 and 2 for these patient populations.

## 5 Conclusions

Our Australian real‐world study demonstrated that the duration of 2L treatment among patients with MM, including those refractory to lenalidomide, increased after DVd received government funding for this indication. This suggests that public access to novel therapies may improve treatment outcomes. To ensure equitable access and maximize the impact of medical innovation, it is essential that new treatments are listed on national formularies (such as the Australian PBS), allowing more patients to benefit from advancements in healthcare.

It is important to continuously evaluate the impact of access to new therapies using real‐world benchmarking. This allows the differences between clinical trial outcomes and real‐world efficacy to be assessed and comparisons of newer therapeutic options with previous standard of care options [26]. These findings underscore the importance of RWE in complementing clinical trial data and informing treatment decisions in routine practice.

## Author Contributions

F.B. was responsible for conceptualization of the study, securing funding project supervision, project administration, and writing the manuscript. S.S. was responsible for the data analysis and writing the manuscript. B.W. was responsible for the data curation, the data analysis, and writing the manuscript. C.P. was responsible for writing the manuscript.

## Funding

This study was funded by Johnson & Johnson Australia and New Zealand. J&J manufactures daratumumab, and contracted Model Solutions to perform the analysis.

## Ethics Statement

The study was approved by the Australian Government Department of Human Services External Request Evaluation Committee (RMS4613).

## Consent

The authors have nothing to report.

## Conflicts of Interest

Felix Buescher, Steve Su, and Cletus Pinto were Janssen‐Cilag Pty Ltd (a Johnson & Johnson company) employees, the sponsor of daratumumab, at the time of this analysis. Ben Waterhouse is the director of Model Solutions, which was funded by Janssen‐Cilag Pty Ltd to undertake this analysis.

## Supporting information




**Supporting Information**: jha270377‐sup‐0001‐figureS1.jpg

## Data Availability

The data that support the findings of this study are available from Department of Health and Services Australia. Restrictions apply to the availability of these data, which were used under license for this study. However, the authors can provide the data upon reasonable request, subject to approval and permission from Department of Health and Services Australia.
